# Dissecting the Physiological Function of Plant Glyoxalase I and Glyoxalase I-Like Proteins

**DOI:** 10.3389/fpls.2018.01618

**Published:** 2018-11-12

**Authors:** Jessica Schmitz, Alessandro W. Rossoni, Veronica G. Maurino

**Affiliations:** ^1^Institute of Developmental and Molecular Biology of Plants, Plant Molecular Physiology and Biotechnology Group, Heinrich Heine University, and Cluster of Excellence on Plant Sciences (CEPLAS), Düsseldorf, Germany; ^2^Institute of Plant Biochemistry, Heinrich Heine University, and Cluster of Excellence on Plant Sciences (CEPLAS), Düsseldorf, Germany

**Keywords:** glyoxalase I, methylglyoxal, scavenging, abiotic stress, reactive carbonyl species, glyoxalase system

## Abstract

The Arabidopsis genome annotation include 11 glyoxalase I (GLXI) genes, all encoding for protein members of the vicinal oxygen chelate (VOC) superfamily. The biochemical properties and physiological importance of three Arabidopsis GLXI proteins in the detoxification of reactive carbonyl species has been recently described. Analyses of phylogenetic relationships and conserved GLXI binding sites indicate that the other eight GLXI genes (GLXI-like) do not encode for proteins with GLXI activity. In this perspective article we analyse the structural features of GLXI and GLXI-like proteins, and explore splice forms and transcript abundance under abiotic stress conditions. Finally, we discuss future directions of research on this topic with respect to the substrate identification of GLXI and GLXI-like proteins and the need of reliable quantitative measurements of reactive carbonyl species in plant tissues.

## Introduction

The glyoxalase (GLX) system was biochemically characterized almost 70 years ago Racker (1951) and is one of the most important lines of defense against glycation in most organisms (Thornalley, 2003; Sousa Silva et al., 2013). The GLX system is a two-step scavenging pathway comprising two phylogenetically unrelated enzymes ultimately detoxifying the reactive dicarbonyl species methylglyoxal (MGO). In a preceding step, MGO is scavenged by reduced glutathione (GSH) forming a hemithioacetal that is the actual substrate for the first reaction catalyzed by GLXI resulting in S-D-lactoylglutathione. In a second step, S-D-lactoylglutathione is converted into D-lactate by the action of glyoxalase II (GLXII), thereby releasing GSH. The action of the GLX system prevents the reaction of free MGO with DNA, lipids, and proteins. MGO reacts preferentially with arginine or lysine residues and any protein with these residues will be prone to glycation. The modified molecules, which are hampered in their functionality are generally called advanced glycation end-products (Sousa Silva et al., 2013).

In Arabidopsis three gene loci encode for active GLXI (Kaur et al., 2013; Jain et al., 2016; Schmitz et al., 2017). These enzymes belong to the group of VOC family proteins, all featuring bidentate coordination of a substrate to a divalent metal center through vicinal oxygen atoms as a common trait (He and Moran, 2011). GLXI use either Ni^2+^/Mn^2+^ or Zn^2+^ for its catalytic activity. Apart from the already characterized GLXI, the remaining eight Arabidopsis proteins fall into the category of GLXI proteins (GLXI-like), due to related structural features. This structural feature, the VOC fold, is determined by two tandem βαβββ modules assigned as single VOC domain (cd06587) forming an incompletely closed, 8-stranded β-barrel containing the catalytic center (He and Moran, 2011). Intriguingly, GLXI and some other VOC family proteins like the bleomycin resistance protein assemble the VOC fold from two monomers exchanging part of their structure to form an intertwined homodimer by a process called domain swapping (Dumas et al., 1994; Bennett and Eisenberg, 2004).

## Homology and Conserved Binding Motifs in Arabidopsis Glyoxalase I and Glyoxalase I-Like Proteins

Recently, we functionally characterized the predominant GLXI isoform involved in MGO detoxification in Arabidopsis, the Zn^2+^-dependent GLXI;3, as well as the Ni^2+^-dependent GLXI;1 and GLXI;2 (Schmitz et al., 2017). All three GLXI isoforms convert MGO and glyoxal using GSH. Analyses of Arabidopsis loss-of-function lines revealed that elimination of toxic reactive carbonyl species during germination and seedling establishment highly depends on the activity of the cytosolic GLXI;3 isoform (Schmitz et al., 2017).

So far, GLXI-like proteins have not been characterized at the molecular level. The ectopic expression of a GLXI-like protein ortholog from *Xerophyta humilis* (DSI;1; desiccation induced 1) in *Escherichia coli* conferred low level of MGO tolerance, leading to the conclusion that DSI;1 homologs are unlikely to have GLXI activity (Mulako et al., 2008). The substrate specificities of all GLXI-like proteins of the VOC superfamily in plants remain unknown. A simple protein blast search in the reference protein database shows that GLXI-like proteins from Arabidopsis have no significant hits (*e* < 10E-13) in the Animalia, Fungi or Archaean group, but are broadly distributed and diverged in Bacteria and Viridiplantae.

**FIGURE 1:**
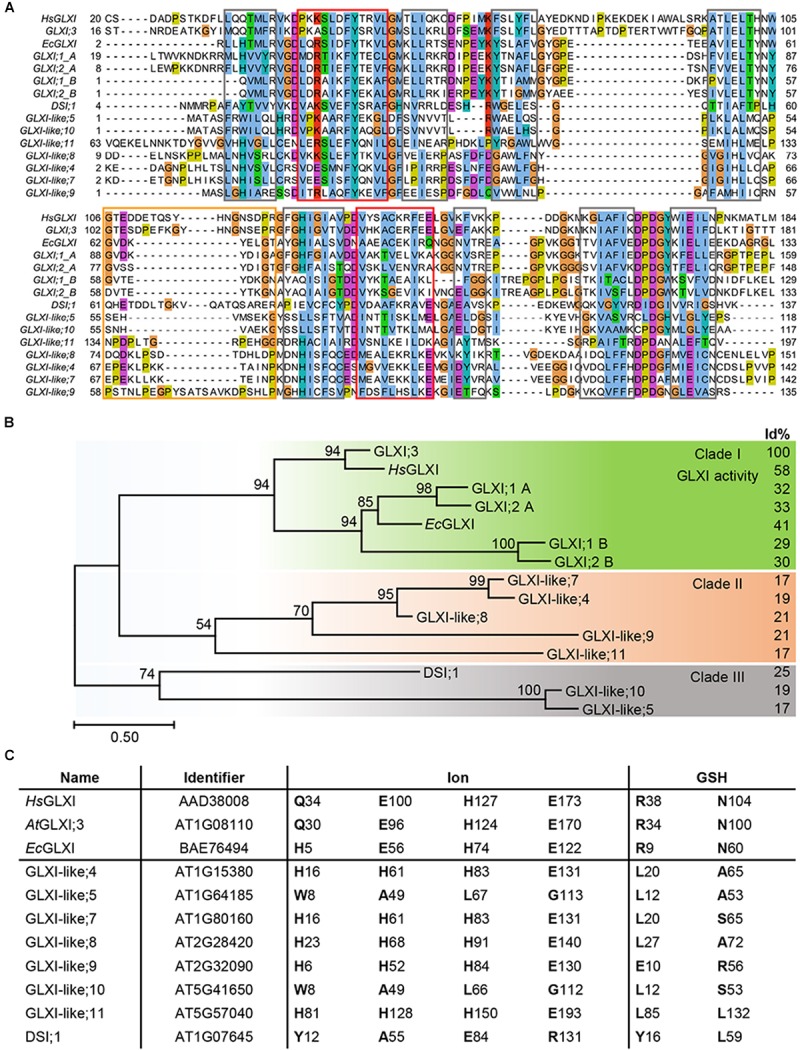
Molecular phylogenetic analysis of Arabidopsis GLXI and GLXI-like proteins. **(A)** Aligned amino acid sequences of GLXI- and GLXI-like proteins. PROMASL3D alignment was constructed with default settings and displayed with Jalview (Pei et al., 2008b; Waterhouse et al., 2009); non-conserved N and C-terminal parts are partially masked. highlighted: gray = β-sheet, red = α-helix, and orange = hinge region. **(B)** Molecular phylogenetic analysis. The evolutionary history was inferred by using the Maximum Likelihood method based on the best fitting Le_Gascuel_2008 model (Le and Gascuel, 2008). The percentage of trees in which the associated taxa clustered together is shown next to the branches based on 1000 bootstrap replicates. A discrete Gamma distribution was used to model evolutionary rate differences among sites [5 categories (+G, parameter = 3.4527)]. The rate variation model allowed for some sites to be evolutionarily invariable [(+I), 1.49% sites]. The tree is drawn to scale, with branch lengths measured in the number of substitutions per site (Scale bar). The analysis involved 15 amino acid sequences. All positions containing gaps and missing data were eliminated. There were a total of 101 positions in the final dataset. Evolutionaryanalyses were conducted in MEGA7 (Kumar et al., 2016). Percent of identical amino acid positions relative to Arabidopsis GLXI;3 (*At*GLXI;3) in relation to the length of a pairwise alignment are displayed to the right (Id%). **(C)** Conserved amino acid positions for GLXI function. Amino acid positions involved in ion or glutathione binding were interfered from the structure-based sequence alignment of Human GLXI (*Hs*GLXI), *Escherichia coli* (*Ec*GLXI), *At*GLXI;3, and compared to the Arabidopsis GLXI-like proteins.

Nine of the 11 Arabidopsis genes of the VOC family encode for single VOC domain proteins, GLXI;3 and all GLXI-like proteins, with molecular sizes of 13–22 kDa. In contrast, the two Ni^2+^-dependent GLXI;1 and GLXI;2 are two-domain (domain A and B) VOC proteins of 33 kDa. While these two-domain Ni^2+^-dependent GLXI fold and function as a monomer, the one-domain GLXI;3 and GLXI-like proteins are likely to assemble as homodimers to reconstitute the VOC fold by domain swapping (He and Moran, 2011; Turra et al., 2015). Multiple sequence alignments (MSA) as well as comparison of conserved GLXI binding sites revealed essential differences in amino acid (aa) composition among the 11 VOC superfamily members. A MAFFT MSA indicated several indels in either the GLXI or GLXI-like proteins, where the closest GLXI homolog DSI;1 shares only 25% identical aa positions with the Zn^2+^-dependent GLXI;3 in relation to the alignment length (Katoh et al., 2005; Sela et al., 2015). All other GLXI-like proteins share 17–21% identity with the predominant Zn^2+^-dependent GLXI;3 indicating a distant relationship of the VOC family members. As the MAFFT algorithm failed to align all βαβββ modules correctly, we tested various alignment programs and found that PROMALS3D (Pei et al., 2008a) performed best in detecting and aligning the secondary structure features that are responsible for the VOC fold (Figure 1A). Based on this MSA, Arabidopsis GLXI and GLXI-like proteins form three distinct clades in a phylogenetic analysis (Figure 1B). Clade I is composed of proteins with proven GLXI activity (Schmitz et al., 2017). Clade II, and Clade III are composed of GLX-like protein for which no experimental evidence of their biological function exist and no close homologs from bacteria have been characterized to date.

Several studies have identified the aa positions responsible for either metal ion or substrate binding and hence for the catalytic activity of the GLXI homologs. In Zn^2+^-dependent GLXI the metal ion binding center within a β-barrel is formed by four essential aa, a glutamine, two glutamic acids, and a histidine (in Human GLXI: Q34, E100, H127, and E173) (Cameron et al., 1997). In Ni^2+^-dependent GLXI the glutamine is exchanged for a histidine (in *E. coli* GLXI: H5, E56, H74, and E122) (He et al., 2000). Even though not the aa but rather an α-structural component determines GLXI metal selectivity (Clugston et al., 2004; Suttisansanee et al., 2015), mutation studies on the ion binding aa clearly confirmed their importance for the catalytic activity (Ridderstrom et al., 1998; Frickel et al., 2001). The aa of the ion binding site are strictly conserved among active Ni^2+^- or Zn^2+^-dependent GLXI proteins but are different within the GLXI-like proteins. In GLXI-like; 4, 7, 8, 9, and 11 the aa at these specific positions are all changed to H, H, H, E, suggesting a similar biochemical property for the GLXI-like proteins of Clade II (Figures 1B,C).

Two conserved arginine and asparagine residues lying in close proximity to the catalytic site in the tertiary structure are responsible for glutathione binding and are highly conserved among GLXI proteins (in Human GLXI: R38, and N104). Furthermore, Cameron et al. (1997) postulated five important conserved aa within a hinge region involved in domain swapping (in Human *Hs*GLXI: G106, Y115, G118, N119, and G124). Interestingly, position G106 is conserved in all GLXI but not in GLXI-like proteins. Actually, the putative hinge region is quite diverse in all VOC family proteins and predicted to have a disordered structure explaining why the other aa Y115, G118, N119, and G124 are not found at the exact positions within the MSA. Notably, none of the Arabidopsis GLXI-like proteins have conserved Zn^2+^ or Ni^2+^ ion, and GSH binding sites suggesting a different biochemical function for the GLXI-like proteins (Figure 1C).

## Expression and Transcriptional Regulation of Glyoxalase I-Like Splice Forms

**FIGURE 2:**
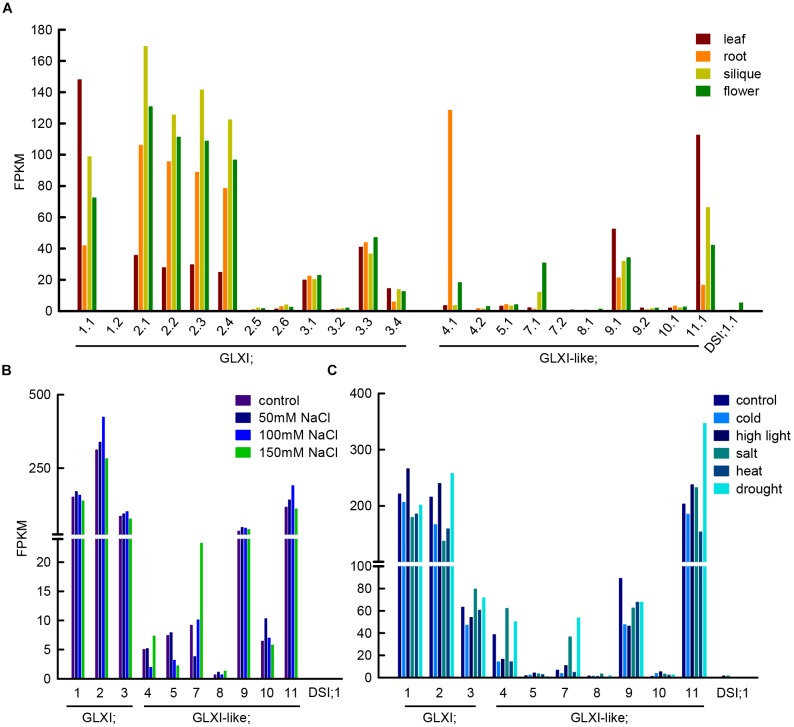
Transcript abundance of GLXI and GLXI-like splice forms and gene expression under abiotic stress conditions. **(A)** FPKM (fragments per kilo base of exon per million fragments mapped) values of GLXI and GLXI-like splice forms in Arabidopsis leaf, root, siliques, and flowers. Raw data was taken from Liu et al. (2012) (PRJNA168212). **(B)** GLXI, and GLXI-like transcript abundance in FPKM in 21-days-old Arabidopsis plants after 24 h treatment with 50, 100, and 150 mM NaCl. Raw data taken from Shafi et al. (2015) (PRJNA217812). **(C)** GLXI, and GLXI-like transcripts in FPKM in whole 12-days-old Arabidopsis seedlings after 24 h of abiotic stress exposure. Raw data taken from Filichkin et al. (2010) (SRA009031). Raw data was re-mapped with high stringency to the TAIR 10 annotation as described in Schmitz et al. (2017).

Methylglyoxal can be produced in several reactions, such as lipid peroxidation, oxidation of aa, and the enzymatic oxidation of ketone bodies, but its main source is the action of triosephosphate isomerase during glycolysis (Semchyshyn, 2014). The enediol phosphate intermediate of the isomerisation can escape from the catalytic center and decompose spontaneously into inorganic phosphate, and MGO, linking in this way, central sugar metabolism and MGO formation. In line with the GLXI function in glycation defense, Arabidopsis GLXI isoforms are highly expressed in heterotrophic as well as autotrophic tissues (Figure 2A) and the transcription of the Ni^2+^-dependent GLXI;2 shows regulation upon alterations in cellular sugar levels (Schmitz et al., 2017). *In silico* analyses of transcriptional responses of GLX to abiotic stress in Medicago, Glycine, Arabidopsis, and Oryza consistently indicated that the expression of GLXI-like homologs is highly modulated by abiotic stresses, while Ni^2+^- and Zn^2+^-dependent GLXI homologs are expressed at a high constitutive level, and show no, or low transcriptional regulation under abiotic stress (Mustafiz et al., 2011; Ghosh and Islam, 2016; Ghosh, 2017).

We stringently re-mapped RNAseq data from Liu et al. (2012) from leaf, root, flower, and siliques, and discriminated between the different splice forms of the GLXI-like proteins in Arabidopsis. We found that only the first of the predicted splice forms is preferentially transcribed (Figure 2A). GLXI-like;4 transcripts are highly abundant in roots, GLXI-like;7 is moderately expressed in flowers, and GLXI-like;9, and 11 are expressed in all tissues tested, with higher expression in leaf. GLXI-like;5, 8, 10, and DSI;1 are extremely low expressed (FPKM < 5) in all organs tested (Figure 2A). Mulako et al. (2008) demonstrated that Arabidopsis DSI;1 mRNA transcripts are found at high levels in mature seeds and are not induced upon desiccation stress in other vegetative tissues. The analysis of transcriptional regulation of GLXI, and GLXI-like proteins in Arabidopsis leaves upon 24 h exposure to different NaCl concentrations (Shafi et al., 2015) shows that the Ni^2+^-dependent GLXI;2 is moderately induced at 100 mM NaCl (Figure 2B). Among the GLXI-like proteins, the homologs 7, 10, and 11 respond to NaCl stress (Figure 2B). However, the induction of expression is not correlated with increasing NaCl concentrations. The analysis of an alternative RNAseq study on 12-day-old seedlings treated during 24 h with different stresses (Filichkin et al., 2010), indicates increase of transcript abundance of the Ni^2+^-dependent GLXI;1, GLXI;2 and of GLXI-like;11 under high light. In this study, the Zn^2+^-dependent GLXI;3, GLXI-like;4, 7, and 11 show induction by 100 mM NaCl. This analysis also shows the induction of the Ni^2+^-dependent GLXI;2, GLXI-like;4, 7, and 11 by drought stress (Figure 2C). A directed and elaborate qPCR-based approach discriminating between the GLXI and GLXI-like gene expression in correlation with abiotic stresses in different plant tissues and stages is needed. This will help dissecting the role of GLXI and GLXI-like proteins in housekeeping and abiotic stress responses.

## Future Challenges and Perspectives

### Identification of Glyoxalase I-Like Substrates

Determination of the substrate specificities is fundamental to dissect the biological functions and to understand the importance of the GLXI and GLXI-like proteins. The actual substrate of GLXI is the hemithioacetal formed by the spontaneous reaction of MGO and GSH. Alternative substrate activities have been shown for glyoxal and phenylglyoxal in the presence of GSH (Vander Jagt et al., 1972; Schmitz et al., 2017). Whether the GLXI-like proteins utilize GSH is unclear. From our present analysis we hypothesize that GLXI-like proteins do not use GSH, as the GSH binding sites are not conserved in these proteins. GLXI use bivalent metal ions as cofactors, these are important for the catalytic activity and in the case of Ni^2+^-dependent GLXI also for substrate specificity (Schmitz et al., 2017). The specific aa involved in metal ion binding in the GLXI proteins are not conserved in GLXI-like proteins, but the majority of the VOC family members need bound metal ions for their catalytic activity (Fe^2+^, Mn^2+^, Zn^2+^, Ni^2+^, or Mg^2+^) (He and Moran, 2011). It is tempting to speculate that the GLXI-like proteins convert other α-keto aldehydes, that might be produced during abiotic stress without using GSH, as in the case of the VOC family member 4-hydroxyphenylpyruvate dioxygenase (Moran, 2014). Developing enzymatic assays with purified proteins for substrate screening should clarify the role of the still uncharacterized GLXI-like proteins (Hüdig et al., 2018).

### Reliable Measurements of Quantitative Methylglyoxal Levels in Plants

Unraveling the physiological significance of GLXI and GLXI-like proteins requires a detailed and precise knowledge of steady state concentrations of reactive carbonyl species, such as MGO, in different plant organs and cell compartments under physiological as well as adverse environmental conditions. Measurement of MGO is hampered by the high reactivity of this compound, the need for a derivatization reaction, and its rather low accumulation levels. Several studies have used different methods for extraction, derivatization and detection in different plant species and conditions. This might explain why quantitative reports on MGO range between 3 nmol⋅gFW^-1^ (Rabbani and Thornalley, 2014) and 100 μmol⋅gFW^-1^ (Yadav et al., 2005a). Due to its reactive nature, MGO extraction procedures might impact on the results and lead to an overestimation of the levels (Sousa Silva et al., 2013; Rabbani and Thornalley, 2014). Taking into account that triose phosphates and glucose are the major sources of MGO in physiological metabolism and that their steady state concentrations in Arabidopsis leaves are ∼50 nmol⋅gFW^-1^ in the case of triose phosphates and ∼1 μmol⋅gFW^-1^ in the case of glucose (Arrivault et al., 2009), free MGO levels far beyond these levels are only possible with a completely abolished GLXI function. Considering a steady state MGO concentration of 3 nmol⋅gFW^-1^, it can be deduced that under physiological conditions the GSH:MGO ratio would be around 100:1 (Krueger et al., 2009; Noctor et al., 2011). Thus, conditions that induce a depletion of the GSH pools would imply a fundamental increase in MGO content.

Each abiotic stress factor, like high light, heat, cold, salt or drought has particular as well as overlapping effects and will perturb metabolism by formation of reactive oxygen species, alteration in osmotic potential or disruption of enzymatic functions. Thalmann et al. (2016) demonstrated the importance of starch degradation during the day to regulate osmotic adjustment and growth upon short term osmotic stress. Hence, increase in soluble sugar content may also increase steady state fluxes through glycolysis and with it MGO formation. Under abiotic stress conditions other sources of MGO, such as lipid peroxidation induced by reactive oxygen species, may become important and would explain the vast number of publications reporting that overexpression of Zn^2+^-dependent GLXI or its expression together with a GLXII can confer tolerance toward general stresses in plants (Veena et al., 1999; Yadav et al., 2005b; Gupta et al., 2018). A reproducible and standardized MGO quantification method as that established by Rabbani and Thornalley (2014) should be used to determine the *in vivo* concentrations of MGO in different stress conditions.

## Concluding Remarks

Even though all GLXI and GLXI-like proteins share the structural features of VOC superfamily proteins, they belong to three distinct clades in a phylogenetic analysis. Through analysis of homology and aa conservation, we found that Arabidopsis GLXI-like proteins do not have GLXI conserved substrate and metal binding sites, and in contrast to GLXI proteins their phylogenetic occurrence seems to be restricted to Bacteria and the green lineage. GLXI expression is high and rather constitutive in different plant organs, whereas expression of GLXI-like;4, 7, and 11 mainly respond to abiotic stresses in our analyses. Quantitative measurements of MGO and other reactive carbonyl species from different plant tissues, in different physiological and abiotic stress conditions using loss-of function mutant lines will support the characterization of GLXI-like proteins and will pinpoint their physiological significance. It seems plausible that GLXI-like proteins diverged in plants to fulfill a different function other than MGO detoxification.

## Author Contributions

JS and VGM analyzed data and wrote the manuscript. JS the performed the phylogenetic analysis. AWR performed the re-mapping of RNAseq data.

## Conflict of Interest Statement

The authors declare that the research was conducted in the absence of any commercial or financial relationships that could be construed as a potential conflict of interest.
